# Genetics of Clusterin Isoform Expression and Alzheimer's Disease Risk

**DOI:** 10.1371/journal.pone.0033923

**Published:** 2012-04-10

**Authors:** I-Fang Ling, Jiraganya Bhongsatiern, James F. Simpson, David W. Fardo, Steven Estus

**Affiliations:** 1 Department of Physiology and Sanders-Brown Center on Aging, University of Kentucky, Lexington, Kentucky, United States of America; 2 Department of Biostatistics, University of Kentucky, Lexington, Kentucky, United States of America; Boston University School of Medicine, United States of America

## Abstract

The minor allele of rs11136000 within *CLU* is strongly associated with reduced Alzheimer's disease (AD) risk. The mechanism underlying this association is unclear. Here, we report that *CLU1* and *CLU2* are the two primary *CLU* isoforms in human brain; *CLU1* and *CLU2* share exons 2–9 but differ in exon 1 and proximal promoters. The expression of both *CLU1* and *CLU2* was increased in individuals with significant AD neuropathology. However, only *CLU1* was associated with the rs11136000 genotype, with the minor “protective” rs11136000T allele being associated with increased *CLU1* expression. Since *CLU1* and *CLU2* are predicted to encode intracellular and secreted proteins, respectively, we compared their expression; for both *CLU1* and *CLU2* transfected cells, clusterin is present in the secretory pathway, accumulates in the extracellular media, and is similar in size to clusterin in human brain. Overall, we interpret these results as indicating that the AD-protective minor rs11136000T allele is associated with increased *CLU1* expression. Since *CLU1* and *CLU2* appear to produce similar proteins and are increased in AD, the AD-protection afforded by the rs11136000T allele may reflect increased soluble clusterin throughout life.

## Introduction

Clusterin (CLU, APOJ) has been implicated in diseases ranging from cancer to Alzheimer's disease (AD) (reviewed in [1], [2], [3], [4]). Although the primary role of clusterin in AD is unclear, *CLU* is implicated in AD by several lines of evidence, including (I) *CLU* mRNA and clusterin protein is increased in AD [5], [6], (ii) clusterin is a component of plaques [4], [5], [7], (iii) clusterin modulates AD-related pathways such as inflammation and apoptosis [1], [8], [9] and (iv) clusterin acts as an amyloid-beta (Aß) chaperone to alter Aß aggregation and/or clearance ([10], [11], reviewed in [4], [12], [13], [14]). The physiologic relevance of *CLU* to AD was confirmed recently when *CLU* single nucleotide polymorphisms (SNP)s were associated with AD risk [15], [16], [17], [18], [19]. Overall, *CLU* genetic variation is essentially unequivocally associated with AD given the robust statistical power of the initial genome-wide association studies and subsequent replication studies [15], [16], [17], [18], [19]. How *CLU* SNPs modulate clusterin to alter AD risk is unknown.

Two *CLU* isoforms, *CLU1* and *CLU2*, have been reported that consist of nine exons and differ only in their first exons and associated proximal promoters; *CLU1 is* predicted to encode a nuclear protein and *CLU2* a secreted protein (reviewed in [20]). Additional reported isoforms include a *CLU* isoform that lacks exon 5 and a *CLU* isoform that lacks exon two, which encodes the leader sequence, resulting in another nuclear clusterin [21], [22]. Here, we investigated the hypothesis that *CLU* isoforms are differentially modulated by AD status and AD-associated SNPs. We identified *CLU1* and *CLU2* as the major *CLU* isoforms in human brain. Quantitative expression studies show that both *CLU1* and *CLU2* are increased in AD but only *CLU1* is associated with rs11136000. Lastly, although *CLU1* and *CLU2* are predicted to produce intracellular and secreted proteins, respectively, immunofluorescence and Western blot studies indicate that *CLU1* and *CLU2* both produce secreted proteins that are similar to those detected in the human brain. Overall, we interpret our results as suggesting that SNP-mediated increases in secreted, soluble clusterin expression may act to reduce AD risk.

## Methods

### Ethics Statement

The work described here was performed with approval from the University of Kentucky Institutional Review Board.

### Cell Culture

SH-SY5Y (human neuroblastoma) and HepG2 (human hepatocellular carcinoma) cells were maintained in Dulbecco's modified Eagle's medium (DMEM) supplemented with 10% fetal bovine serum, 50 U/ml penicillin and 50 µg/ml streptomycin at 37°C in a humidified 5% CO_2_ - 95% air atmosphere.

### *CLU* Expression Plasmid

Expression plasmids encoding *CLU1* and *CLU2* were generated from SH-SY5Y cellular mRNA that was reverse transcribed by using the primer 5′-TAGGTGCAAAAGCAACAT-3′ which corresponds to sequence just after the *CLU* stop codon. *CLU1* and *CLU2* cDNAs were then amplified by PCR with forward primers 5′-TGAGTCATGCAGGTTTGCAG-3′ (*CLU1*) and 5′-ATGATGAAGACTCTGCTGCTG-3′ (*CLU2*) used in combination with the common reverse primer 5′-CTCCTCCCGGTGCTTTTTG-3′. PCR fragments were ligated into pcDNA3.1/V5-His-TOPO T/A cloning vector (Invitrogen, Carlsbad, CA). Clones encoding *CLU1* and *CLU2* were detected by PCR screening and clone integrity confirmed by sequencing.

### Human Autopsy Tissue

De-identified human brain specimens were provided by the University of Kentucky AD Center Neuropathology Core [23], [24]. AD and non-AD designations followed NIARI neuropathology guidelines, which include indices of neuritic senile plaques and neurofibrillary tangles, and provide a likelihood staging of AD neuropathological diagnosis [25], [26]. Individuals with “low” AD neuropathology were cognitively intact prior to death and had no or low likelihood of AD by NIARI criteria; their average age at death was 81.8±10.2 (mean ±SD, n = 17). Individuals with “high” AD neuropathology represented a combination of demented individuals with high likelihood of AD by NIARI criteria (n = 27) and cognitively intact individuals that were found to have moderate or high AD neuropathology at death (n = 7); their average age at death was 81.9±6.2 (mean ±SD, n = 34). The average post-mortem interval (PMI) for low AD neuropathology individuals was 3.0±0.8 hours (mean ± SD, n = 17) while the PMI for high AD neuropathology individuals was similar (3.2±0.8 hours (n = 34)). Choroid plexus samples were from six individuals with an average age at death of 80.0± 3.3 years and PMI of 2.9±1.1 hours. Fetal tissue RNA samples were obtained commercially (Stratagene, Santa Clara, CA) and have been described previously [27].

### PCR Amplification

Total RNA was extracted from human brain specimens and converted to cDNA in 1 µg aliquots with random hexamers and reverse transcriptase (SuperScript III, Invitrogen), essentially as we described previously [24], [28], [29], [30]. PCR primers were designed such that the splicing of each internal *CLU* exon as well as *CLU1* and *CLU2* were evaluated (Table 1). In initial screening, cDNA pooled from five high AD neuropathology and five low AD neuropathology samples were subjected to PCR-amplification (Platinum Taq, Invitrogen) by using each primer pair and a PCR profile consisting of initial denaturation for 5 minutes at 95°C, followed by 27–32 cycles of 94°C for 30 s, 60°C for 30 s, and 72°C for 1 min, and final extension at 72°C for 7 min (Perkin Elmer 9600). PCR products were separated by polyacrylamide gel electrophoresis, stained with SYBR Gold and visualized by using a fluorescence imager (Fuji FLA-2000). The identity of the PCR products was confirmed by direct sequencing (Davis Sequencing, Davis, CA).

**Table 1:** PCR Primers for evaluating splice variation.

Exons Amplified	Product Size	Primer	Primer Sequence
1a-5(For *CLU1*)	596 bp	Exon 1a Forward	GCGAGCAGAGCGCTATAAAT
		Exon 5 Reverse	GATGCGGTCACCATTCATC
1b-5(For *CLU2*)	567 bp	Exon 1b Forward	AGATGGATTCGGTGTGAAGG
		Exon 5 Reverse	GATGCGGTCACCATTCATC
4–6	598 bp	Exon 4 Forward	AGAGTGTAAGCCCTGCCTGA
		Exon 6 Reverse	AGACAAGATCTCCCGGCACT
5–9	675 bp	Exon 5 Forward	GGACATCCACTTCCATAGCC
		Exon 9 Reverse	ACTTGGTGACGTGCAGAGC

### Real-time PCR

The expression level of *CLU1* and *CLU2* was quantified by real-time PCR. Each isoform was specifically amplified by using a sense primer corresponding to sequence within their respective exon 1, i.e., 5′-GCGAGCAGAGCGCTATAAAT-3′ for *CLU1* and 5′-AGATGGATTCGGTGTGAAGG-3′ for *CLU2*′, and an antisense primer corresponding to sequence at the exon 2–3 boundary, i.e., 5′-GACATTTCCTGGAGCTCATTG-3′. Note that the CLU1 sense primer contains the TATA sequence suggested by Schepeler et al to be present only in a longer “*CLU34*” isoform [31]. The 20 µl real-time PCR mixture containing approximately 20 ng of brain cDNA, 1 µM of each primer and 1× PerfecTaq PCRSupermix (Quanta Biosciences, Gaithersburg, MD) was subjected to real-time PCR (Bio-Rad, Hercules, CA). PCR profiles consisted of pre-incubation at 95°C for 2 min, followed by 40 cycles of 95°C for 15 s, 60°C for 30 s, and 72°C for 20 s. Specificity of the reactions was evaluated by showing a single PCR product by gel electrophoresis and by performing a melting curve analysis after PCR amplification. The PCR product copy number in each sample was determined relative to standard curves that were amplified in parallel and were based upon previously purified and quantified PCR products. The copy numbers were then normalized to the geometric mean of the copy numbers of hypoxanthine-guanine phosphoribosyltransferase 1 and ribosomal protein L13A as described (Vandesompele et al., 2002; Zhang et al., 2005). All real-time PCR assays were repeated twice.

### Genotyping

DNA samples were genotyped for rs11136000 by using unlabeled PCR primers and TaqMan FAM and VIC dye-labeled MGB probes (Pre-designed SNP Genotyping Assay, Applied Biosystems, Foster City, CA) on a real-time PCR machine (Bio-Rad, Hercules, CA).

### Statistical Analysis

Variation in *CLU* isoform expression were analyzed as a function of rs11136000 genotype, AD neuropathology, sex and age by using a general linear model (SPSS v.18 (IBM, Somers, NY)). A dominant mode of inheritance was assumed to maximize statistical power.

### Immunofluorescence

*CLU* expression plasmids were transfected into SH-SY5Y cells by using FuGene HD Transfection Reagent as directed by the manufacturer (Roche Applied Sciences, Indianapolis, IN). Briefly, 3.75×10^4^ cells/well were maintained in a poly-_L_-lysine treated 8-well chamber coverglass (Lab-Tek, Nunc, Rochester, NY) and transfected the next day with *CLU1* or *CLU2* expression plasmid. Twenty-four hours after transfection, cells were washed with phosphate buffered saline (PBS) and fixed with ice-cold methanol for 5 minutes. Non-specific antibody binding sites were blocked by incubating the cells with 5% goat serum in PBS with 0.1% Tween-20 (PBST) for 1 hour and the cells were then incubated with mouse anti-V5 antibody (1∶1000 dilution, ab27671, Abcam, Cambridge, MA) and either rabbit anti-calnexin antibody (1∶200 dilution, SPA-860, Stressgen, Victoria, BC, Canada) or rabbit anti-TGN46 antibody (1∶1000 dilution, ab16052, Abcam) in 5% goat serum at 4°C overnight. The next day, cells were washed with PBST three times and incubated with a mixture containing Alexa-488 goat anti-mouse IgG antibody (1∶200 dilution, Molecular Probes, Carlsbad, CA) and Alexa-568 goat anti-rabbit IgG antibody (1∶200 dilution, Molecular Probes) in 5% goat serum at room temperature for an hour. After washing with PBST, cells were stained with 10 µg/ml of Hoechst 33258 (Molecular Probes) for 30 minutes, washed with PBS, and subjected to fluorescence microscopy.

### Western Blot

SH-SY5Y cells or HepG2 cells were grown in a 100 mm dish (2.6×10^6^ cells/dish) for 24 hours and then transfected with *CLU1* or *CLU2* expression plasmid by using FuGene HD Transfection Reagent as directed by the manufacturer (Roche Applied Sciences). Twenty-four hours after transfection, cell medium was replaced with Opti-MEM (Invitrogen) and cells maintained for another 24 hours. The conditioned medium was collected and the cells washed with 5 ml of room-temperature PBS. Cells were then lysed in 0.5 ml of RIPA butter (50 mM Tris, pH 8.0, 150 mM NaCl, 1% NP-40, 0.5% deoxycholic acid, 0.1% SDS) containing 1× protease inhibitor cocktail (Roche Applied Sciences) for 30 minutes on ice with occasional rocking. Cell lysates were centrifuged at 10,000×g for 10 minutes at 4°C and the supernatant collected. Conditioned medium was centrifuged at 250×g for 10 minutes and 0.5 ml of the supernatant was collected and supplemented with 1× protease inhibitor cocktail (Roche Applied Science). Human brain anterior cingulate tissue samples were prepared by homogenizing ∼0.5 mg tissue in RIPA buffer supplemented with 1× protease inhibitor cocktail, centrifuging at 14,000×g for 10 minutes at 4°C, and then collecting the supernatant. After the protein concentration of each sample was determined (Micro BCA Protein Assay Reagent Kit, Pierce, Rockford, IL), 15 or 20 µg protein were mixed with SDS sample loading buffer containing β-mercaptoethanol, boiled for 5 minutes and subjected to polyacrylamide gel electrophoresis on a 7.5% polyacrylamide gel. Proteins were transferred to nitrocellulose membranes (Bio-Rad). The blots were then incubated with 5% nonfat dry milk for 1 hour at room temperature and probed overnight with mouse anti-V5 antibody (1∶5000 dilution, Abcam) or mouse anti-clusterin antibody (1∶200, B-5 Santa-Cruz) at 4°C. After washing with PBST four times for 5 minutes each, the blots were incubated with peroxidase-conjugated sheep anti-mouse antibody (1∶1,000 dilution, Jackson ImmunoResearch) for 1 hour at room temperature. Bound peroxidase was visualized by using a SuperSignal West Pico kit (Pierce) and a molecular imager (ChemiDoc XRS System, Bio-Rad). For studies involving PNGase F digestion, each protein sample was treated with PNGase F as directed by the manufacturer (New England Biolabs, Ipswich, MA). Briefly, 15 µg protein of each sample was denatured at 100°C for 10 minutes and incubated with 1,500 units of PNGase F at 37°C for 2 hours. Samples were then analyzed by Western blot as described above.

## Results

**Figure 1:**
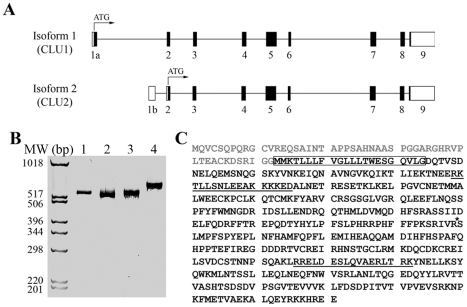
*CLU1* and *CLU2* are the primary *CLU* isoforms expressed in human brain. The exon layout for the *CLU1* and *CLU2* isoforms is shown (A). Coding and non-coding exonic regions are represented by black and white boxes, respectively, while introns are shown as thin black lines. The first ATG site in each isoform is shown as well. Evaluation of *CLU* splicing by RT-PCR identified only *CLU1* and *CLU2* in human brain cDNA (B), i.e., single PCR products are observed upon amplification from exon 1a – exon 5 (lane 1), exon 1b – exon 5 (lane 2), exon 4 – exon 6 (lane 3) and exon 5 – exon 9 (lane 4). Molecular weight markers are shown at the left. The predicted protein sequences encoded by *CLU1* and *CLU2*, beginning with the initial ATG sites shown in A, are shown in panel C. The sequence in gray font is unique to *CLU1* while sequence in black font is common to *CLU1* and *CLU2*. The boxed amino acid sequence represents signal sequence that is removed from clusterin-2 upon its translocation to the ER. Two nuclear localization sequences, as predicted by PSORT II (http://psort.ims.u-tokyo.ac.jp/form2.html), are underlined. The asterisk shows the clusterin cleavage site that separates the α- and β- subunits.

To begin to evaluate *CLU* expression, we screened human brain cDNA to identify *CLU* isoforms present in brain. Previously reported isoforms include *CLU1* and *CLU2*, which are identical in exons 2–9 but differ in exon 1, as well as isoforms lacking exons 2 or 5 ([21], [22], reviewed in [20]). RT-PCR analyses of pooled human brain cDNA samples detected only *CLU1* and *CLU2* (Figure 1A–1B). *CLU2* translation is predicted to result in a secreted protein, beginning at the initial ATG in exon 2 that is common to both *CLU2* and *CLU1* (Figure 1C). In contrast, *CLU1* translation is predicted to initiate at an ATG within its exon 1, which is in-frame with the ATG site in the common exon 2. Hence, *CLU1* is predicted to encode a protein identical to that encoded by *CLU2* except that the *CLU1* protein would contain 52 additional amino acids at its amino terminus (Figure 1C). This change is predicted by PSORTII to result in an intracellular and likely nuclear, form of clusterin (reviewed in [20]). In summary, *CLU1* and *CLU2* are the primary *CLU* isoforms in brain and are predicted to encode intracellular and secreted proteins, respectively.

**Figure 2:**
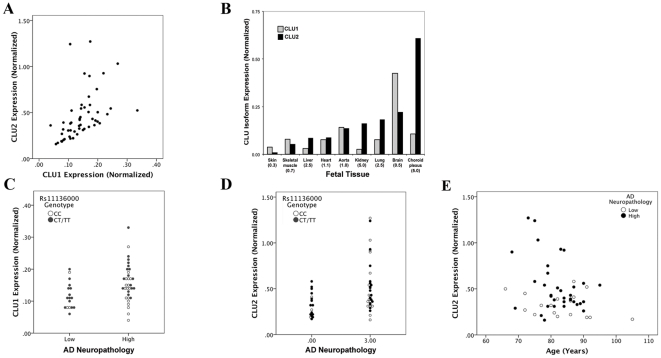
Expression of *CLU* isoforms in human tissues. *CLU1* and *CLU2* isoforms were quantified by isoform-specific RT-PCR in a series of adult human cingulate and choroid plexus samples (A–B) as well as a human fetal tissues (B). Within A, each marker reflects the expression of *CLU1* and *CLU2*, normalized to housekeeping genes, in a single brain sample. Within B, the value in parentheses following the tissue name is the *CLU1*∶*CLU2* ratio. *CLU1* expression shows a positive association with AD neuropathology and rs11136000T allele (C). *CLU2* expression was increased in individuals with AD pathology and decreased with age (D–E).

To evaluate whether rs11136000, the primary AD-related *CLU* SNP [15], [16], [17], [18], [19], is associated with *CLU* expression, we quantified *CLU1* and *CLU2* expression in a series of brain samples. The *CLU* isoforms were quantified in separate real-time PCR assays that used forward primers corresponding to their unique exon 1 and a common reverse primer that targeted the exon 2–3 boundary. Copy numbers were determined relative to a standard curve for each isoform and normalized to the geometric mean of housekeeping genes [23], [32]. The expression of *CLU1* and *CLU2* were modestly coordinately regulated (Figure 2A), with CLU2 being consistently greater than CLU1; the overall *CLU2*∶ *CLU1* ratio was 3.35±1.84 (mean ± SD, n = 51). To gain further insights into *CLU* isoform expression, we quantified *CLU1* and *CLU2* in a series of single human fetal tissue samples as well as a set of six adult choroid plexus samples (Figure 2B). Among the fetal tissues, the *CLU2*∶ *CLU1* ratio varied widely from 0.3 (skin) to 6.2 (kidney) (Figure 2B). The *CLU2∶CLU1* ratio in choroid plexus, which produces cerebrospinal fluid, was 5.72±0.65 (mean ± SD, n = 6).

We next evaluated *CLU* isoform expression as a function of AD neuropathology, rs11136000 status, sex and age. We found that *CLU1* expression was significantly increased with high AD neuropathology and the minor rs11136000T allele (Figure 2C, Table 2). In contrast, *CLU2* expression was significantly increased with AD neuropathology but not rs11136000 genotype, and decreased with age (Figure 2D–E, Table 3). Overall, both *CLU1* and *CLU2* expression was increased with AD, confirming prior reports. However, only *CLU1* expression was associated with rs11136000. Since the minor rs11136000T allele was associated with both increased *CLU1* expression and reduced AD risk [15], [16], [17], [18], [19], we interpret these results as suggesting that increased *CLU1* expression is associated with reduced AD risk.

**Table 2:** Analysis of *CLU1* Expression.

Parameter	Coeff.	SE	P value
AD Neuropathology	0.01675	0.00551	0.004
Rs11136000 (dominant model)	0.01927	0.00798	0.020
Sex	−0.00708	0.01503	0.640
Age	−0.00112	0.00104	0.286

*CLU1* expression was analyzed as a function of rs1136000, AD neuropathology, sex and age by using a general linear model. This model assumed a dominant mode of inheritance for the SNP. The estimated marginal means for *CLU1* in rs11136000 CC versus CT/TT individuals were 0.112±0.013 and 0.151±0.010 (mean ± SE), respectively. The estimated marginal means for low and high AD neuropathology were 0.106±0.014 and 0.157±0.009 (mean ±SE), respectively. These values were modeled with age equal to 82.3 years.

**Table 3:** Analysis of *CLU2* Expression.

Parameter	Coeff.	SE	P value
AD Neuropathology	0.06482	0.02483	0.012
Rs11136000 (dominant model)	0.00697	0.03594	0.847
Sex	0.03399	0.06766	0.618
Age	−0.01024	0.00467	0.033

*CLU2* expression was analyzed as a function of rs1136000, AD neuropathology, sex and age by using a general linear model. A dominant mode of inheritance was assumed for rs11136000. The estimated marginal means for low and high AD neuropathology were 0.332±0.062 and 0.526±0.041 (mean ±SE), respectively. These values were modeled with age equal to 82.3 years.

**Figure 3:**
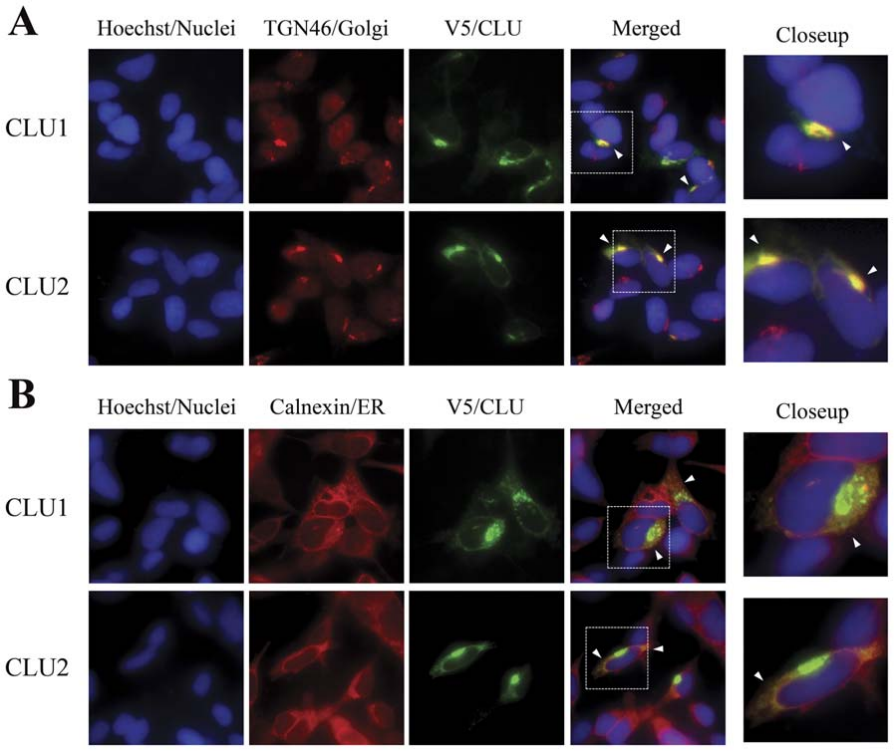
*CLU1* and *CLU2* encoded proteins are localized to the ER and Golgi. SH-SY5Y cells were transfected with vectors encoding *CLU1* and *CLU2*; the resultant clusterin expression was localized by a vector-derived V5 epitope tag. The subcellular localization for both proteins overlapped with the Golgi, as shown by co-labeling with antibody against TGN46 (A) and with the ER, as shown by co-labeling with an antibody against calnexin (B).

*CLU1* and *CLU2* are predicted to encode intracellular and secreted proteins, respectively (Figure 1). Therefore, we evaluated the proteins produced by *CLU1* and *CLU2* transfected cells to discern whether these corresponded to the intracellular and secreted forms of clusterin that have been reported [4], [8], [33]. When each isoform was transiently transfected into neural SH-SY5Y cells, immunofluorescence analyses showed a similar subcellular localization pattern (Figure 3). The proteins produced by *CLU1* and *CLU2* tended to concentrate within the Golgi apparatus, as established by double-labeling with an antibody against trans-Golgi network protein 46 (TGN46) (Figure 3A). Relatively modest amounts of clusterin were also detected within the endoplasmic reticulum (ER), as established by double labeling with calnexin (Figure 3B). Nuclear, cytosolic, or mitochondrial-associated clusterin was not observed. Hence, the clusterin produced by *CLU1* and *CLU2* manifests an intracellular localization that includes the ER and Golgi, consistent with a possible secretory pathway for both proteins.

**Figure 4:**
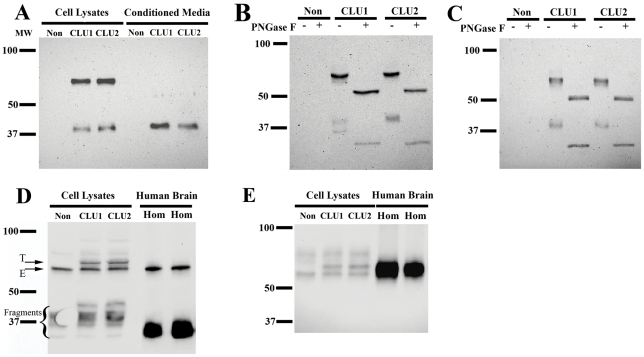
*CLU1* and *CLU2* produce clusterin proteins that are similar to each other and to clusterin in human brain. Transfection of *CLU1* and *CLU2* resulted in proteins of similar size, appearing as both intact and cleaved clusterin in cell lysates and conditioned media; the proteins resulting from *CLU* transfection were detected via their V5 epitope tag (A–B). PNGase F treatment prior to electrophoresis reduced the size of the proteins produced by *CLU1* and *CLU2* similarly (B). The apparent doublet in the protein encoded by *CLU1* at 37 kDa was variable between experiments and likely reflects incomplete glycosylation as the doublet collapsed to a single band after PNGase F digestion. Results similar to those depicted in B were obtained when *CLU1* and *CLU2* were transfected into HepG2 cells (C). When transfected and endogenous clusterin proteins were detected with an antibody raised against a carboxyl-terminal clusterin epitope (D–E), intact clusterin from transfection (T-labeled arrow) was slightly larger than endogenous clusterin (E-labeled arrow), reflecting the ∼5.1 kDa V5 epitope and His tags in the ectopic clusterin (D). The bands labeled “Fragments” are clusterin alpha subunits derived from variably glycosylated endogenous and transfected clusterin. Overall, the cellular clusterin proteins are similar in size to the human brain clusterin, as discerned for the intact and cleaved clusterin under reducing conditions (D) or as the heterodimeric clusterin under non-reducing conditions (E).

*CLU1* is predicted to encode a 501 amino acid protein with a molecular weight of 57.8 kDa while *CLU2* is predicted to encode 449 amino acids, totaling 52.5 kDa. During maturation of secreted clusterin, the protein is N-glycosylated and proteolyzed to generate a heterodimer that is maintained by disulfide bonds [20]. To compare the size of the proteins produced by *CLU1* and *CLU2*, we performed Western blot analyses of transfected SH-SY5Y cells; ectopic clusterin was detected via a V5 epitope tag (Figure 4 A–B). Cell lysates contained both intact and cleaved clusterin while conditioned medium contained only cleaved clusterin; the clusterin protein produced by *CLU1* and *CLU2* appeared equal in size (Figure 4A). To discern whether glycosylation differences may obscure differences in the clusterin molecular weight derived from their primary sequence, the protein mixtures were digested with PNGase F to remove N-linked sugars prior to electrophoresis; the size of the proteins produced by *CLU1* and *CLU2* transfected cells were both decreased similarly by PNGase treatment (Figure 4B). We considered the possibility that *CLU* translation may show cell-type specificity, and therefore repeated this study in an unrelated cell line by transfecting *CLU1* and *CLU2* into HepG2 cells. As with the SH-SY5Y cells, *CLU1* and *CLU2* transfected cells produced clusterin of similar size, even after PNGase F digestion (Figure 4C). Hence, we found no evidence to suggest that the proteins produced by *CLU1* and *CLU2* differ in cellular localization, size, glycosylation, or proteolytic cleavage. Rather, when these Western blot studies are considered in combination with the immunofluorescence studies, *CLU1* and *CLU2* appear to produce equivalent proteins that are similarly translated, glycosylated, and cleaved as they pass through the ER and Golgi in the secretory pathway to the extracellular space.

To evaluate whether the clusterin proteins produced by *CLU1* and *CLU2 in vitro* are similar to those produced *in vivo*, we compared ectopic clusterin with endogenous clusterin in SH-SY5Y cells and in human brain by Western blotting with a clusterin antibody. Endogenous clusterin in SH-SY5Y cells migrates at a slightly lower molecular weight than that resulting from *CLU* transfection, reflecting the 5.1 kDa V5/His tag encoded by the pcDNA3.1 vector expressing transfected *CLU* (Figure 4D). Importantly, this 5.1 kDa size difference between endogenous and transfected clusterin is similar to the size difference between the *CLU1* and *CLU2*-predicted proteins, indicating that if the proteins encoded by *CLU1* and *CLU2* differed by 5 kDa, the proteins would be resolved easily under these experimental conditions. The endogenous SH-SY5Y clusterin migrates similarly to the intact protein detected in brain homogenates suggesting these cellular forms are similar to those present *in vivo* (Figure 4D). To extend this analysis to the heterodimeric form of clusterin, we also performed electrophoresis under non-reducing conditions. We found that heterodimeric protein from *CLU1* and *CLU2* transfected cells appeared similar in size to each other and to the forms found in brain (Figure 4E). Hence, *CLU1* and *CLU2* produce proteins *in vitro* that are similar in size to endogenous clusterin in vitro and human brain.

## Discussion

The main findings of this report are several. First, *CLU1* and *CLU2* are the primary *CLU* isoforms in human brain and differ in their first exon and proximal promoter. Second, the expression of only *CLU1* was associated with rs11136000 while both *CLU1* and *CLU2* were increased with robust AD neuropathology. Third, *CLU1* and *CLU2* produce secreted clusterin proteins that are similar to each other and to clusterin in human brain. Since *CLU* is increased in AD and *CLU1* is increased with the minor rs11136000T allele, which is also associated with reduced AD risk, we interpret our results as suggesting that increased *CLU* expression throughout life may reduce AD risk. Overall, these results identify a possible means underlying the association of rs11136000 with AD risk.

The association between *CLU* expression and rs11136000 was discerned because the *CLU* isoforms were analyzed separately. For example, when we analyzed total *CLU* expression in the same fashion as reported here, total *CLU* was not associated with rs11136000 (p = 0.51) although a robust association with AD pathology was observed (p = 0.005, Ling et al., unpublished observations). The lack of a significant association between rs11136000 and total *CLU* is somewhat disconcerting since *CLU1* and *CLU2* appear to produce a similar protein. We speculate that (i) the threshold for clusterin biological significance may be less than the threshold for overall *CLU* statistical significance and/or (ii) *CLU1* may be enriched in a cell type different than *CLU2*, which may affect its functionality. We note that the lack of an association between total *CLU* and rs11136000 was similar to prior reports that total *CLU* mRNA or clusterin protein were not associated with rs11136000 [17], [34]. As the clusterin produced by *CLU1* and *CLU2* appears similar, discerning an association between rs11136000 and clusterin would be especially challenging in tissues with a large proportion of *CLU2*. In this regard, *CLU2* was the major isoform in choroid plexus, suggesting that an association between cerebrospinal fluid clusterin and rs11136000 would be difficult to detect. The differential regulation of *CLU1* and *CLU2* was not unexpected because *CLU1* and *CLU2* have separate proximal promoter regions. Consistent with this observation, Cochrane et al found that androgen-treatment in a prostate cancer cell line decreased *CLU1* expression while enhancing *CLU2* expression [35]. Although the identification of the functional SNPs and transcription factors that modulate *CLU* expression is beyond the scope of this report, we note that rs11136000 resides within intron 3 and is common to both *CLU1* and *CLU2*. Hence, rs11136000 is likely not functional but rather is in linkage with a functional SNP more proximal to the *CLU1* promoter. In summary, the association between rs11136000 and *CLU1* was dependent upon analyzing the two *CLU* isoforms separately.

Since earlier predictions were that *CLU2* encodes a secreted protein while the additional 52 amino-terminal residues encoded by *CLU1* would result in its intracellular localization [20], we expected clusterin in *CLU1* and *CLU2* transfected cells to differ in cellular locale and size. In support of this prediction, the ATG translation initiation sites in the *CLU1*-specific exon 1a as well as the common exon 2 both satisfy Kozak consensus sequence requirements (http://bioinfo.iitk.ac.in/AUGPred/). However, clusterin in *CLU1* and *CLU2* transfected cells was localized to the secretory pathway and was equivalent in size under conditions that would easily discern their predicted five kDa size difference, especially after PNGase F treatment to remove sugar residues. Hence, *CLU1* and *CLU2* produce similar secreted proteins *in vitro*. Comparable results were obtained in two cell types, suggesting that *CLU1* and *CLU2* generally produce soluble clusterin protein. The most parsimonious interpretation of these data is that *CLU1* translation is generally initiated at the common exon 2 ATG. The possibility exists that a portion of *CLU1* translation is initiated at the exon 1a ATG at levels too low to be readily detected or that produce an unstable protein which is rapidly degraded. Distinguishing among these possibilities is a future direction for this work. Overall, we summarize our current results by noting that the proteins produced from *CLU1* and *CLU2 in vitro* are similar to each other, and similar in size to those present in human brain, leading us to interpret our data as supporting the possibility that *CLU1* and *CLU2* generally produce secreted clusterin protein *in vitro* and *in vivo*.

A logical extension of the finding that AD risk is reduced with a genetic variant that correlates with increased *CLU* expression is that other factors that increase *CLU* expression may also reduce AD risk. Furthermore, since *CLU* expression is increased in AD without reversing the disease, we speculate that enhanced *CLU* expression reduces AD risk only if *CLU* expression is increased well before AD onset, mimicking the likely SNP effects. One possible modulator of interest is age-dependent changes in sex hormones since *CLU* isoforms are differentially regulated by androgens [35]. Stress has also been shown to upregulate *CLU* in many instances [36] and may cause the AD-associated increase in *CLU* observed here. In terms of possible therapeutic agents, multiple histone deacetylase inhibitors increase *CLU* expression robustly *in vitro* [37]. Interestingly, one member of this class, valproate, increases *CLU* expression in human astrocytes [37] and reduces amyloid accumulation as well as behavioral deficits in mouse amyloid models [38], [39]. While valproate may also act via other mechanisms [38], the actions of valproate on *CLU* expression may be relevant to its neuroprotection and merit further exploration to reduce AD risk.

Limitations of this study include that rs11136000 and AD neuropathological status capture only ∼20% of the variance in *CLU* expression. Identifying the unknown factors that account for the additional variance, which could include epigenetic factors, will facilitate understanding of *CLU* expression. Moreover, variability in these factors could facilitate or obscure the SNP association with *CLU1* expression described here, suggesting that replication of these data in additional samples is necessary for their acceptance by the research community. Additionally, since the nuclear form of clusterin arising from an isoform lacking exon 2 has been associated with apoptosis [21], [22], this isoform may be transiently expressed and hence below our limits of detection, especially in comparison with *CLU1* and *CLU2*.

In conclusion, the AD-protective allele of rs11136000 was associated with increased expression of *CLU1* but not *CLU2* in our dataset. Both *CLU1* and *CLU2* produce a soluble, secreted clusterin protein that is similar to that observed in human brain. Discerning the association between rs11136000 and *CLU1* depended upon analyzing these two CLU isoforms separately.
